# Spatial and temporal characteristics of vegetation phenology in the middle and lower reaches of the Yangtze River and its response to climate

**DOI:** 10.3389/fpls.2025.1589908

**Published:** 2025-06-19

**Authors:** Zhang Shikang, Zhang Wei, Li Xiaotong

**Affiliations:** ^1^ College of Resources and Environment, Anhui Science and Technology University, Fengyang, China; ^2^ College of Architecture, Anhui Science and Technology University, Bengbu, China; ^3^ College of Management, Anhui Science and Technology University, Bengbu, China

**Keywords:** middle and lower reaches of the Yangtze River, vegetation phenology, spatial and temporal analysis, climate change, growing season

## Abstract

This study investigates the response mechanism of vegetation phenology to climate change in the middle and lower reaches of the Yangtze River from 2001 to 2022, aiming to reveal the spatial and temporal evolution patterns of vegetation SOS, EOS, and LOS and their driving factors, and to provide a scientific basis for regional ecological management. Based on the EVI dataset, climate parameters were extracted by S-G filtering and dynamic thresholding method and combined with one-way linear regression, stability analysis, and partial correlation analysis to assess the vegetation climate changes and their responses to air temperature, precipitation, sunshine hours, and surface temperature. The results showed that: (1) SOS advanced overall (0.29 d/a), EOS delayed (0.26 d/a), and LOS prolonged (0.56 d/a). (2) Significant trends of SOS advance and EOS postponement were observed in coniferous forests, agricultural fields, and natural vegetation, and EOS advance was significant in broadleaf forests. (3) In the future, SOS and EOS will continue to advance, and LOS of cropland will continue to extend. (4) Air temperature, precipitation, and sunshine hours have an advancing effect on SOS, surface temperature has a postponing effect on EOS, and precipitation and surface temperature have an extending effect on LOS. Vegetation climate change is affected by the complex interaction of climate factors, and the results of the study reveal its spatial and temporal evolution patterns and response mechanisms to climate change, providing an important reference for regional ecological assessment and management.

## 1 Introduction

Vegetation phenology pertains to the timing of periodic events in the plant life cycle, such as germination, flowering, and defoliation, and their interactions with environmental factors, including temperature, precipitation, and light (White et al., 2002). As a fundamental component of ecosystems, vegetation phenology serves as an indicator of plant adaptation to climate change and plays a crucial role in regulating the global carbon cycle, water cycle, and energy balance (Richardson et al., 2013; Yuke et al., 2024). In recent years, the intensification of global warming has led to its widespread application in studies assessing the impacts of climate change on ecosystems, making it a focal area of research in ecology (Ziang et al., 2023), climatology, and environmental science (Piao et al., 2019).

The selection of study areas is critical in identifying the spatial and temporal characteristics of vegetation phenology and its response to climate change. In temperate and boreal regions, for instance, climate change has a substantial impact, and alterations in vegetation phenology can significantly influence ecosystem structure and function (Menzel et al., 2006). However, due to the spatial differences in climatic background, vegetation type and intensity of human activities, the response mechanism of physical climate shows significant regional specificity. This spatial heterogeneity requires the selection of study areas to consider the typicality and diversity to provide a differentiated strategy for regional ecological management. Pirone et al. (Pirone et al., 2024). found that ignoring reservoir-channel dynamic interactions overestimates flood control effectiveness. In Lama and Errico’s study (Lama and Chirico, 2020; Errico et al., 2019), vegetation phenology affected hydrodynamic modeling by changing stem properties, which helped prioritize different management strategies. The analysis can quantify the cascading effects of the climate-climate-hydrology system, thus determining the thresholds of influence of the critical period of vegetation phenology on the system, affecting the design of flood control, wetland management, and other practical applications. Furthermore, the response mechanism of vegetation phenology varies spatially due to differences in climatic conditions, vegetation types, and the intensity of human activities across geographic regions (Cleland et al., 2007). The study identified the mechanisms driving diurnal temperature and precipitation differentiation and their elevation/vegetation type dependence on phenology, providing a region-specific basis for optimizing carbon cycle models and ecosystem management (Ma et al., 2022; Shen et al., 2023). Consequently, selecting representative study areas facilitates an understanding of general patterns in vegetation phenology and offers a scientific foundation for regional ecosystem management and climate adaptation strategies.

Research has shown that in the northern hemisphere at middle and high latitudes, spring phenology generally occurs earlier, while fall phenology is delayed, resulting in an extended growing season (Gao et al., 2019). However, variations in regional and vegetation type responses to climate change remain significant. For example, in arid and semi-arid regions, vegetation phenology exhibits higher sensitivity to precipitation changes than to temperature variations (Meng et al., 2020). Investigating the response mechanism of vegetation phenology to climate change remains a central research focus. Vegetation generally exhibits an asymmetric response to key phenological periods such as leaf spreading, flowering, and fruiting as temperatures rise, precipitation and other patterns change. While spring warming typically advances the growing season, fall cooling delays its end (Piao et al., 2006). Temperate woody plants tend to diverge with warming in their flowering and leaf-spreading intervals due to reduced winter vernalization and increased cumulative temperature demand (Guo et al., 2023). Additionally, other climatic factors, such as precipitation and light, play crucial roles in vegetation phenology. Drought stress, in particular, inhibits plant growth and influences phenological shifts (Ren et al., 2017), a phenomenon that has been exacerbated by global warming driven by increased greenhouse gas emissions (Zhang et al., 2024). In addition, urbanization further disturbs phenological rhythms through the heat island effect and land-use change. These changes not only affect species adaptations but also have the potential to reshape ecosystem functioning through a chain reaction of carbon cycling and water-heat balance (Zhao and Wu, 2013).

Vegetation phenology directly affects the hydrological function of vegetation through the beginning or end of the growing season and seasonal changes in leaf area, and (Lama and Crimaldi, 2021) showed that vegetation morphology parameters such as density, height, and LAI significantly regulate the hydrodynamic processes in the river channel and floodplain. Accurate monitoring of vegetation phenology provides dynamic input parameters for hydrological models, and the hydraulic model (Lama et al., 2020) and flow resistance quantification method (Walter et al., 2021) in the literature can improve the simulation ability of vegetation-flow feedback mechanism, thus enhancing the prediction accuracy of the hydrological and ecological processes in the middle and lower reaches of the Yangtze River. Remote sensing technology overcomes spatial constraints, enabling more effective monitoring of vegetation phenology by providing phenology parameters at various scales for detailed analysis. Studies utilizing remote sensing datasets, such as MODIS and AVHRR, have demonstrated a trend of an extended growing season in the Northern Hemisphere, attributing this to the advancement of spring phenology and the delay of fall phenology as primary driving factors (Piao et al., 2015; Zhang et al., 2003). Furthermore, integrating ground observation data with model simulations has facilitated a deeper exploration of the intrinsic relationship between vegetation phenology and climate variables, including temperature and precipitation (Chen et al., 2018).

Despite advancements in this field, certain limitations persist. Compared to traditional climate monitoring methods, remote sensing data, while covering extensive regions, possess limited spatial and temporal resolution, making it difficult to detect climate variations at finer scales (Tang et al., 2016). Additionally, significant variations exist among vegetation types and geographic regions, with most research focusing on temperate and boreal zones, whereas tropical and subtropical regions remain relatively understudied (Gonsamo et al., 2021). Moreover, the influence of human activities, such as land-use changes and urbanization, on vegetation phenology has not been extensively investigated, constraining a comprehensive understanding of phenology-driving factors (Zhou et al., 2014).

The normalized difference vegetation index (NDVI) is commonly employed for extracting vegetation phenology through remote sensing by establishing temporal growth curves of vegetation (Huete et al., 2002). However, once vegetation cover reaches a certain threshold, NDVI tends to saturate, limiting its effectiveness (Liu et al., 2018). Sunlight-induced chlorophyll fluorescence has emerged as a novel approach for vegetation phenology monitoring, yet its application at finer scales is restricted by spatial resolution constraints (Guanter et al., 2012; Joiner et al., 2016). (Li et al., 2010) highlighted that the EVI2 index, derived from two spectral bands, offers advantages such as an extended time series, reduced interference from soil background effects, and resistance to saturation, allowing for better monitoring of vegetation changes in areas with dense vegetation cover (Jiang et al., 2008). Lan et al. (2025) also found that the EVI data were more accurate, which is slightly affected by the soil and the atmosphere conditions.

This study utilized EVI data from 2001 to 2022 to extract vegetation phenology parameters in the middle and lower reaches of the Yangtze River through the dynamic threshold method. The spatial and temporal distribution, as well as the trends of different vegetation phenology parameters, were analyzed, along with their relationship with meteorological factors. The middle and lower reaches of the Yangtze River represent one of the most densely populated and economically active watersheds in China, experiencing significant environmental pressures (Xu et al., 2024). Understanding the spatial and temporal characteristics of vegetation phenology and its response to climate factors provides a scientific foundation for regional ecosystem management and conservation. The findings of this study offer valuable insights into mitigating the adverse effects of climate change through adjustments in land-use practices and vegetation management strategies within the framework of climate change adaptation.

## 2 Materials and methods

### 2.1 Study area

**Figure 1:**
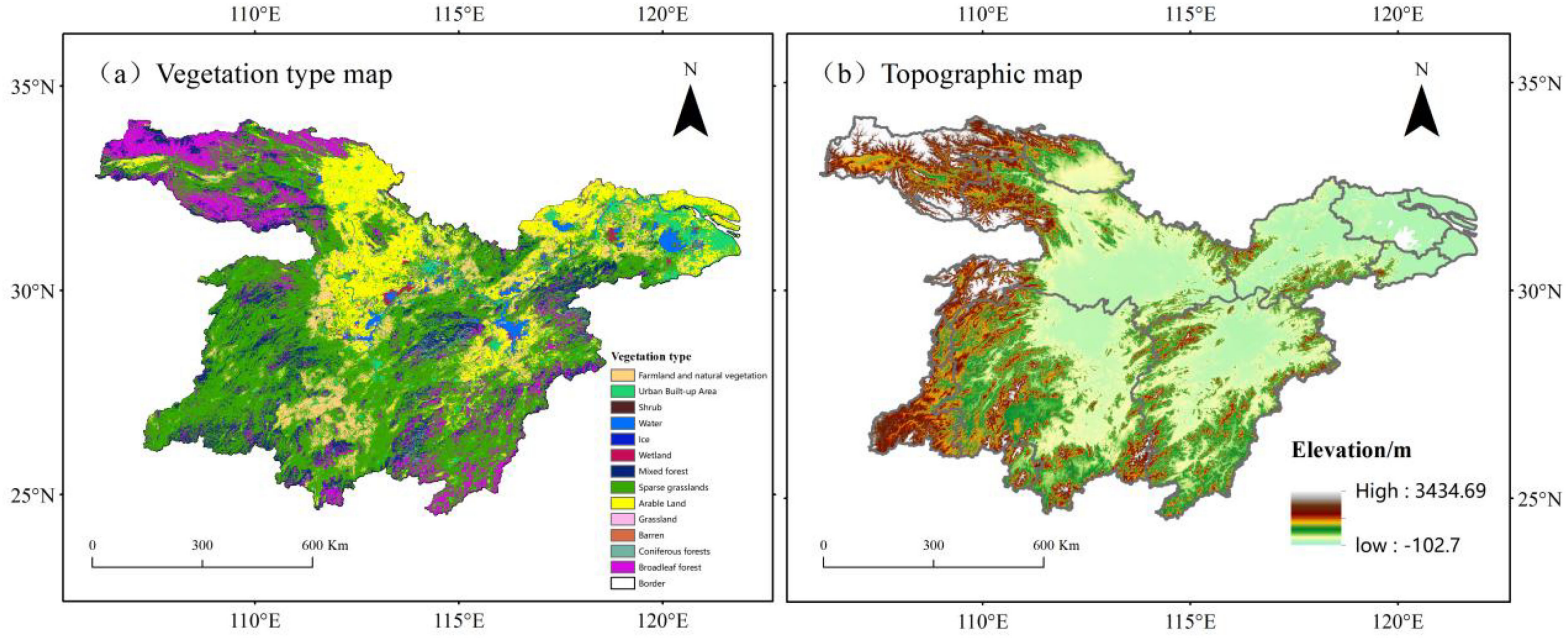
**(a)** Representation of different colors in the legend to represent different vegetation types in the study area. **(b)** Representation of DEM in the middle and lower reaches of the Yangtze River.

The middle and lower reaches of the Yangtze River extend from the Three Gorges Reservoir area to the river’s mouth, encompassing multiple provinces in eastern and central China. This region spans 16 provinces and municipalities, covering a watershed area of about 743,600 square kilometers. Geographically, it is situated between longitudes 106°12′58″–122°25′ and latitudes 24°34′15″–34°11′37″. The terrain is primarily composed of hills and plains, with diverse vegetation types. It serves as a significant agricultural and industrial hub and is among the most densely populated areas in China (**Figure 1**).

### 2.2 Data sources

#### 2.2.1 Study area data

The dataset utilized in this study consists of the 1:1 million boundary data (2009) for the Middle and Lower Yangtze River Basin. Data sources are detailed in **Table 1**. To ensure consistency in data integration, the spatial projection coordinate system was standardized to WGS_1984_UTM_Zone_49N, and all resolutions were resampled to 250 m.

**Table 1:** Sources and applications of data.

Data	Duration	Source	Use
Enhanced Vegetation Index(EVI)	2001–2022	LP DAAC-MOD13Q1	Extraction of vegetation phenology parameters
Study area boundary data	2009	(http://www.geodata.cn)	Definition of the extent of the study area
temperatures(Temp)	2001–2020	GRDC(www.gis5g.com)	Monthly average temperature data
precipitation(Prcp)	2001–2020	CHG/CHIRPS/DAILY	Monthly average precipitation data
surface temperature(LST)	2001–2020	ECMWF v5(ERA5)	Monthly mean surface temperature extraction
daylight hours(SD)	2001–2020	GRDC(www.gis5g.com)	Average annual cumulative sunshine hours
Land use data	2020	LP DAAC-MCD12Q1	Definition of different vegetation types

#### 2.2.2 Remote sensing data

The Enhanced Vegetation Index (EVI) was selected as the primary data source, featuring a spatial resolution of 250 m and a temporal resolution of 16 days. All data processing and downloads were conducted using the Google Earth Engine (GEE) platform. EVI, a second vegetation index with higher sensitivity to areas of high biomass, was employed in this study for vegetation monitoring.

#### 2.2.3 Climate data

Climate data, including precipitation (Prcp) and surface temperature (LST), were obtained from the European Centre for Medium-Range Weather Forecasts (ECMWF) atmospheric reanalysis dataset. These data have a spatial resolution of 0.1° and cover the period from January 1950 to the present (ERA, 2017). Additionally, air temperature (Temp) data were sourced from the China 1 km-resolution annual mean air temperature dataset, with a spatial resolution of 0.0083333° (~1 km) for the period 2001–2022.

#### 2.2.4 Land use data

The MODIS land cover product, based on the International Geosphere-Biosphere Program (IGBP) classification scheme, was utilized (Hansen et al., 2000). This product categorizes global vegetation into 17 types, from which eight were selected for this study based on regional conditions. These include farmland and natural vegetation mosaic, urban built-up areas, sparse grassland, steppe, cropland, coniferous forest, broadleaf forest, and mixed coniferous-broadleaf forest. Other land cover types, such as shrublands, were not considered due to their limited spatial extent. The dataset has a spatial resolution of 500 m and an annual temporal resolution.

### 2.3 Research methodology

#### 2.3.1 Phenological extraction

In this study, three climatic parameters—SOS (the start of the growing season), EOS (the end of the growing season), and LOS (the length of the growing season)—were selected to assess the response of vegetation phenology to climate variations. Vegetation phenology parameters were extracted using the dynamic threshold method, while the EVI time series curves for the middle and lower reaches of the Yangtze River from 2001 to 2022 were fitted using TIMESAT 3.3 and the S-G filter in Matlab.

The thresholds for the onset and end of vegetation phenology were set at 20%, and the primary extracted phenological parameters included the vegetation greening period, yellowing period, and growing season length. These parameters enabled the characterization of vegetation phenology in the middle and lower reaches of the Yangtze River over the study period from 2001 to 2022. The MODIS-EVI dataset was derived through a series of preprocessing steps, including cropping, de-clouding, reprojection, and resampling, based on the GEE MODIS dataset (MOD13Q1.006). The S-G filtering formula applied in the analysis was as follows **Equation 1**:


(1) $${\text{Y}}_{\text{j}}^{*}=\frac{\sum_{\text{i}=-\text{n}}^{\text{n}}{\text{C}}_{\text{j}}{\text{Y}}_{\text{j}+1}}{2\text{n}+1}$$

In the formula, Yj+1 represents the j-th initial value of the sliding window, corresponding to the smoothed EVI data. Cj denotes the filter coefficient derived from the least squares fitting, while 2n+1 represents the size of the sliding window.

#### 2.3.2 Trend analysis

Linear regression analysis was employed to examine variations in phenological indicators from 2001 to 2022, using the following calculation formula **Equation 2**:


(2) $$\text{Slope}=\frac{\sum_{\text{i}=1}^{\text{n}}\left(\text{i}\times {\text{y}}_{\text{i}}\right)-\sum_{\text{i}=1}^{\text{n}}\text{i}\sum_{\text{i}=1}^{\text{n}}{\text{y}}_{\text{i}}}{\text{n}\sum_{\text{i}=1}^{\text{n}}{\text{i}}^{2}-{\left(\sum_{\text{i}=1}^{\text{n}}\text{i}\right)}^{2}}$$

Here, i represents the year, n is set to 22, and SOSi, EOSi, and LOSi refer to the respective phenological parameters in year i. The slope describes the temporal trend of these climatic parameters in units of days per year (d/a). A negative slope indicates a shortening trend in LOS, with SOS and EOS shifting earlier. The magnitude of the slope determines the rate at which SOS and EOS advance or delay and the extent of LOS changes over time.

#### 2.3.3 Partial correlation analysis and T-test

The correlation between variables is typically represented by the correlation coefficient R. When analyzing the relationship between vegetation phenology and specific climatic variables, the influence of other climatic factors—such as average temperature, average precipitation, sunshine hours, and surface temperature—must be controlled. In order to eliminate the effects of covariates among multiple influencing factors, this study utilized Pearson correlation analysis to investigate the correlation between vegetation phenology parameters and five factors, including climatic factors, at the metric scale, following the formula **Equation 3**:


(3) $${\text{R}}_{\text{xy}}=\frac{\sum_{\text{i}=1}^{\text{n}}\left(\begin{array}{c} {\text{x}}_{\text{i}}-\bar{\text{x}} \end{array}\right)\left(\begin{array}{c} {\text{y}}_{\text{i}}-\bar{\text{y}} \end{array}\right)}{\sqrt{\sum_{\text{i}=1}^{\text{n}}{\left(\begin{array}{c} {\text{x}}_{\text{i}}-\bar{\text{x}} \end{array}\right)}^{2}}\sqrt{\sum_{\text{i}=1}^{\text{n}}{\left(\begin{array}{c} {\text{y}}_{\text{i}}-\bar{\text{y}} \end{array}\right)}^{2}}}$$

where, i represents the year, and n is set to 20. The variables denote temperature, precipitation, sunshine hours, and surface temperature, in relation to vegetation phenology. The mean values of these variables and vegetation phenology indicators are represented by X and Y, respectively. The correlation coefficient R ranges from [-1,1], where a negative R indicates an inverse correlation. This suggests that an increase in meteorological factors leads to an advancement in SOS, a delay in EOS, and an extension of LOS. Conversely, a positive R implies the opposite effect. If P<0.05, the correlation is considered significant; if P>0.05, the correlation is not statistically significant.

#### 2.3.4 Stability analysis

The coefficient of variation (CV) was used to analyze changes in phenological indicators at the pixel scale from 2001 to 2022. The calculation method is as follows **Equation 4**:


(4) $$\text{CV}=\delta /\bar{x}$$

Here, the standard deviation and mean of SOS, EOS, and LOS are represented by σ and µ, respectively. When the mean value approaches zero, the data exhibit unstable variations. Based on observed conditions, CV was categorized into five levels: (1) CV<0.2, low fluctuation; (2) 0.2 ≤ CV< 0.4, relatively low fluctuation; (3) 0.4 ≤ CV< 0.6, moderate fluctuation; (4) 0.6 ≤ CV< 0.8, relatively high fluctuation; and (5) CV ≥ 0.8, high fluctuation.

#### 2.3.5 Continuity analysis

The Hurst index (H) is a statistical indicator used for time series analysis and is effective in predicting the stability of future trends. The value of H ranges from 0 to 1, with its magnitude reflecting the persistence strength of vegetation phenology. The classification of H values follows three scenarios:

1. When H is between 0.5 and 1, vegetation phenology parameters in the middle and lower reaches of the Yangtze River exhibit positive persistence, indicating a correlation between future development trends and historical patterns.
2. When H equals 0.5, the time-series changes in vegetation phenology show no significant correlation between future trends and past variations.
3. When H falls between 0 and 0.5, vegetation phenology exhibits negative persistence, suggesting that future trends will develop in the opposite direction to past changes.

Based on this classification, vegetation phenology parameters in the study area were categorized into four types: strong damaging persistence (0.15 ≤ H< 0.35), weak damaging persistence (0.35 ≤ H< 0.5), weak positive persistence (0.5< H< 0.65), and strong positive persistence (H ≥ 0.65). To enhance understanding and prediction of persistence in future trends, H values for the climatic time series were further classified into nine categories to establish the relationship between future vegetation cover development trends and historical changes in the middle and lower reaches of the Yangtze River.

## 3 Results

### 3.1 Spatial distribution of vegetation phenology

**Figure 2:**
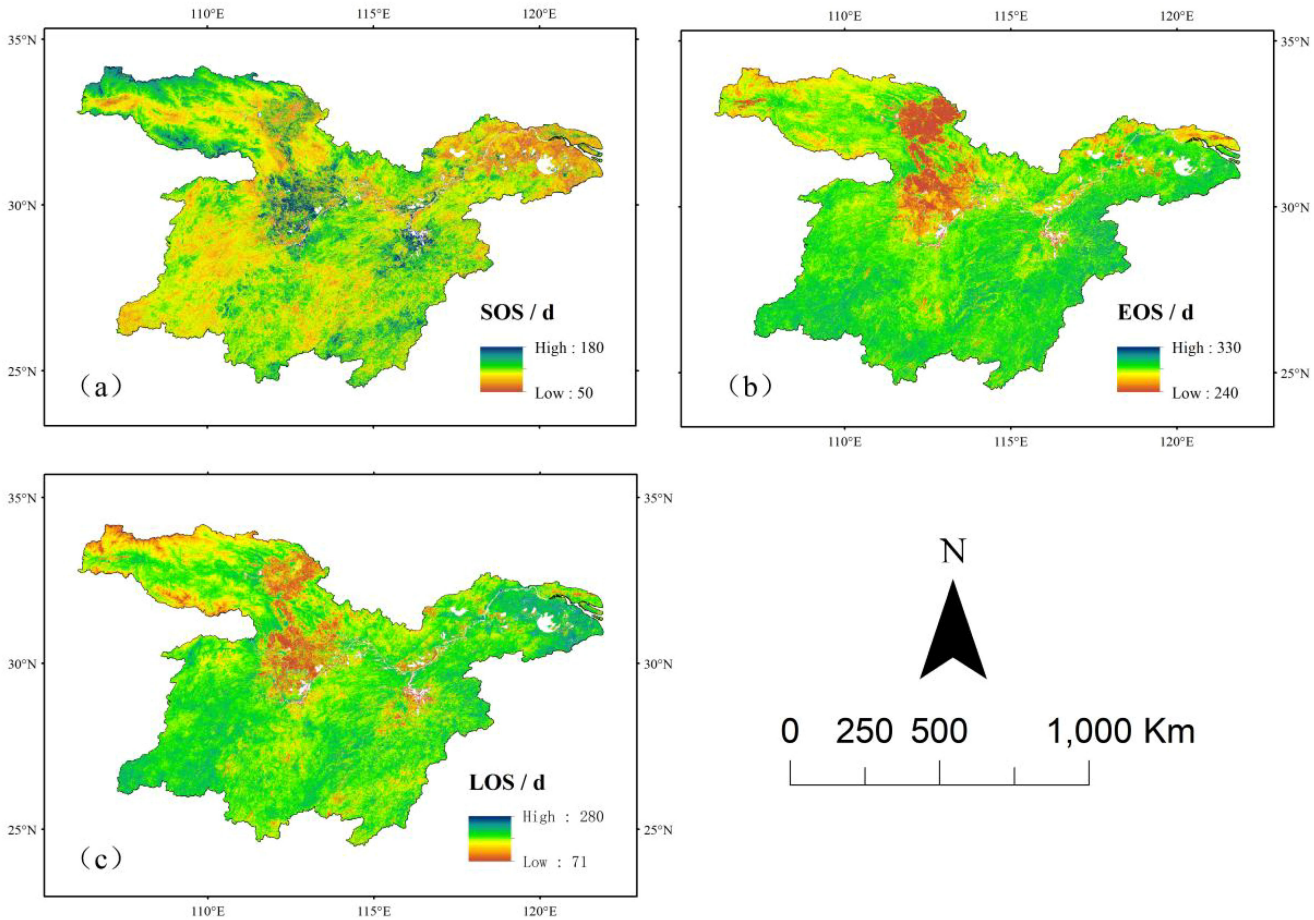
Distribution of vegetation phenology means in the middle and lower reaches of the Yangtze River. **(a)** Mean value distribution of sos. **(b)** Mean value distribution of eos. **(c)** Mean value distribution of los.

**Figure 3:**
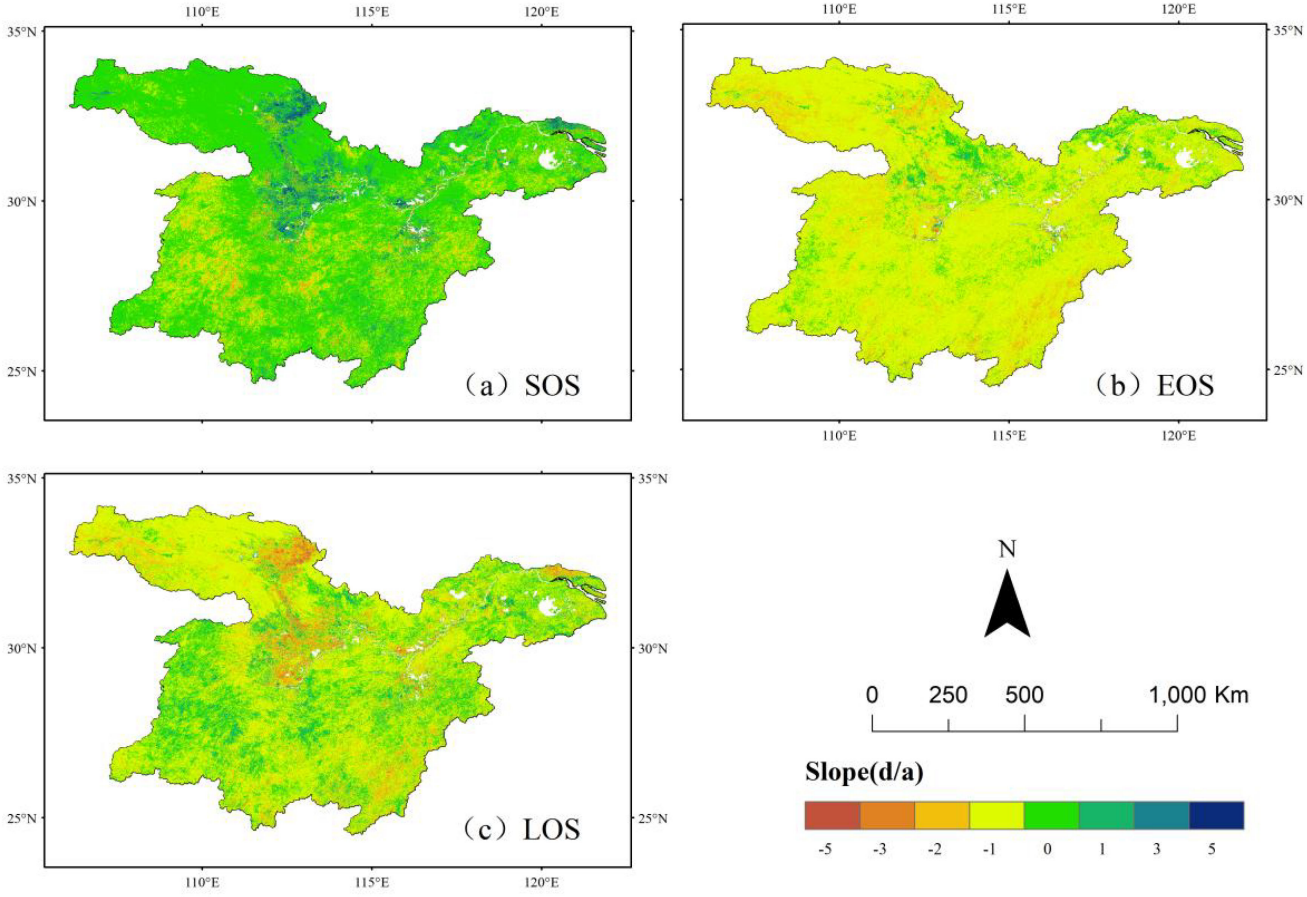
Trend analysis of vegetation phenology in the middle and lower reaches of the Yangtze River. **(a)** Trend analysis of sos. **(b)** Trend analysis of eos **(c)** Trend analysis of los.

The results presented in **Figures 2**, **3** indicate that the start of the growing season (SOS) follows an earlier trend from northwest to southeast, primarily occurring between 62 and 97 days. The SOS in the northeastern region is significantly earlier compared to other areas. Overall, the phenological return period of vegetation in the study area exhibited an advancing trend, with the exception of certain localized regions in the central, northwestern, and southern parts, where vegetation growth showed a delayed trend. The end of the growing season (EOS) is predominantly concentrated between 281 and 315 days, indicating that EOS generally occurs between mid-October and mid-November. The spatial distribution of EOS follows a pattern of higher values in the north and lower values in the south. Earlier EOS occurrences were observed in the northwestern and northeastern parts of the study area, while all other regions exhibited a delayed trend.

The length of the growing season (LOS) is generally distributed between 206 and 243 days. The northwestern region exhibits a relatively shorter LOS, whereas the northeastern and southwestern regions experience longer LOS values. A general trend of LOS extension was observed across the study area, except for the northwestern and central regions, where a shortening trend was detected.

Unlike the distribution of changes in the EOS, variations in the SOS and LOS were not uniform, and significant spatial heterogeneity in vegetation phenology was observed. From 2001 to 2022, the changes in vegetation phenology in the middle and lower reaches of the Yangtze River aligned with the findings of Hong Xinxie et al (Hong and Sun, 2023), demonstrating a trend of advancing SOS, delaying EOS, and an overall lengthening of the growing season.

### 3.2 Spatial and temporal variation in vegetation phenology

**Figure 4:**
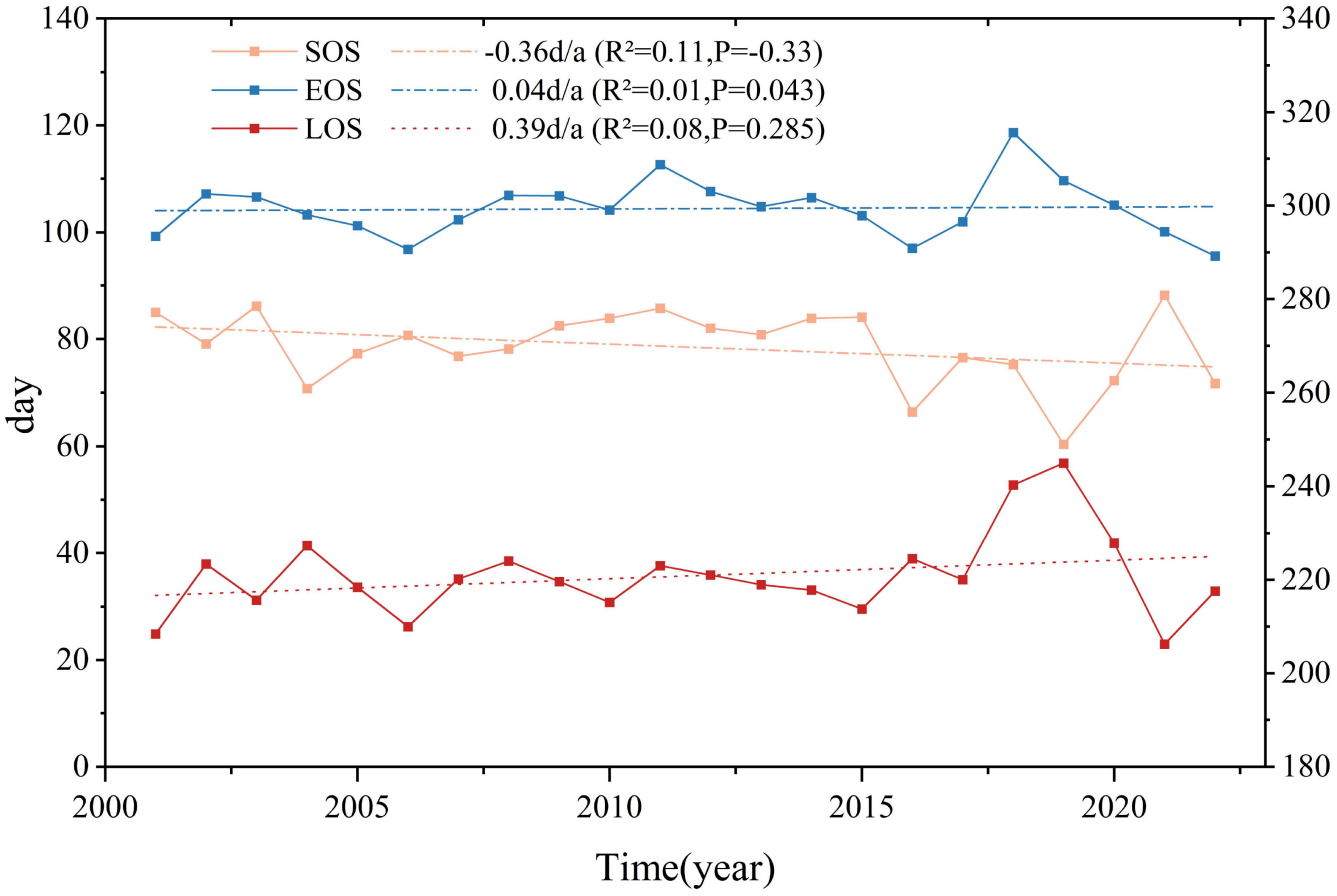
Annual variation trend of vegetation phenology in the middle and lower reaches of the Yangtze River.

The temporal and spatial variations in vegetation phenology, as illustrated in **Figure 4**, exhibit a distinct trend of change over time in the middle and lower reaches of the Yangtze River.

The magnitude of SOS changes remained relatively uniform (p>0.05), with an average advancement of 0.29 days per year. EOS changes, however, demonstrated a significant delaying trend (p<0.01), with an average postponement of 0.26 days per year. Since LOS is defined as the duration between SOS and EOS, the advancing trend of SOS combined with the delayed EOS resulted in an overall extension of LOS. The study findings indicate that from 2001 to 2022, LOS increased at an average rate of 0.56 days per year (p<0.01). The maximum recorded variations were 27.82 days for SOS, 24.89 days for EOS, and about 38.73 days for LOS. The latest SOS and the shortest LOS were observed in 2021, while a relatively extended LOS was recorded in 2018.

**Figure 5:**
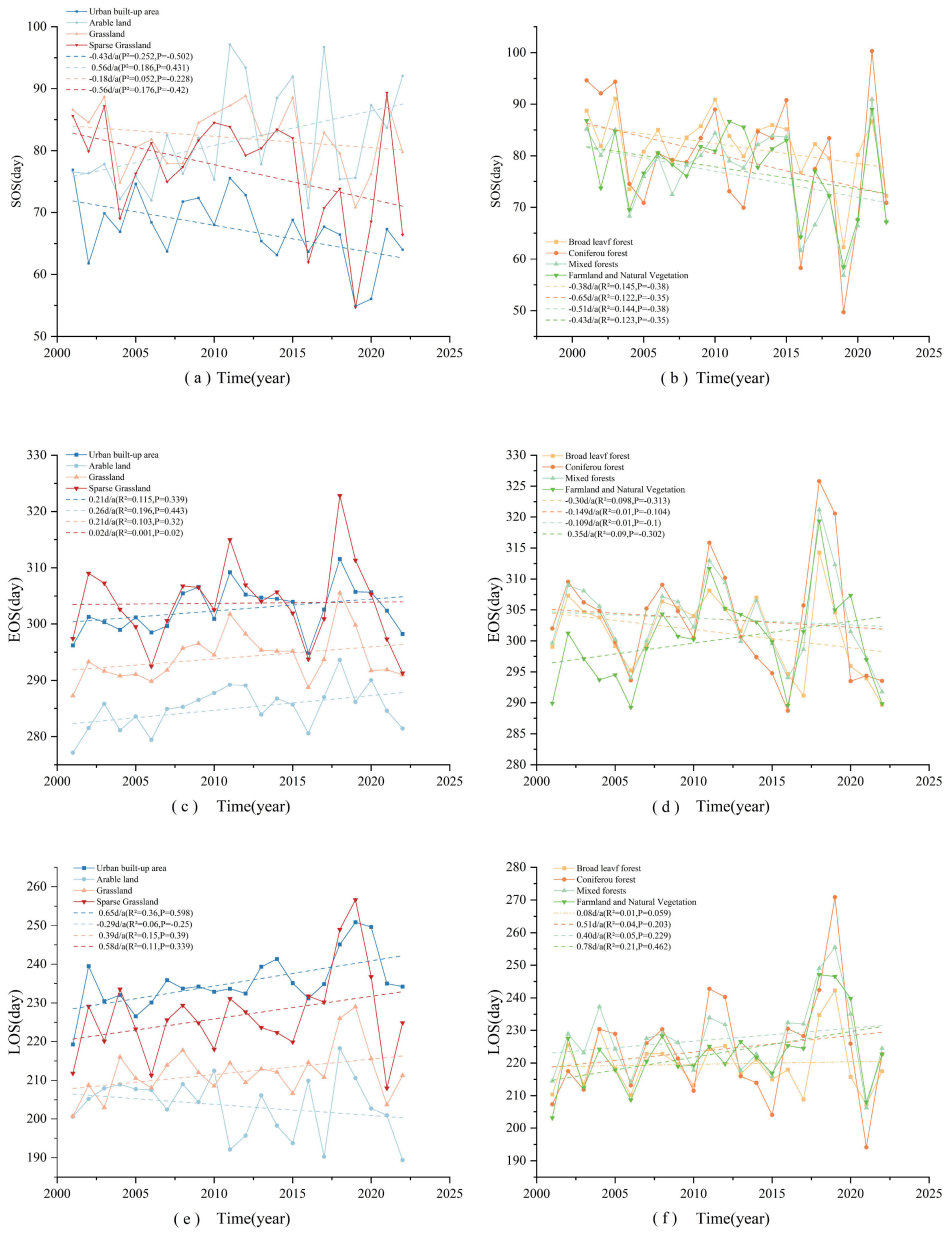
Interannual variability of different vegetation phenology in the middle and lower reaches of the Yangtze River. **(a, c, e)** represents the interannual changes of sos, eos, and los in urban built-up areas, cropland, grassland, and sparse grassland, respectively. **(b, d, f)** represent the interannual variations of sos, eos, and los in broad-leaved forests, coniferous forests, mixed forests, farmlands, and natural vegetation mosaics, respectively.

To examine the phenological changes among different vegetation types in the middle and lower reaches of the Yangtze River, the average phenological values were extracted for urban built-up areas, croplands, grasslands, sparse grasslands, broad-leaved forests, coniferous forests, mixed coniferous and broad-leaved forests, farmlands, and natural vegetation mosaics. Based on an analysis of the long-term time-series data from 2001 to 2022, the spatial and temporal evolution of phenological parameters for various vegetation types in the study area was systematically assessed. Through trend analysis and fitting, a dynamic map depicting the 22-year vegetation phenology period within the study area was generated, along with corresponding quantitative fitting results (**Table 2**; **Figure 5**).

**Table 2:** Relationship between vegetation phenology and various vegetation types in the middle and lower reaches of the Yangtze River.

Vegetation type	Parameters	Simultaneous equations	R^2^	Vegetation type	Parameters	Simultaneous equations	R^2^
Urban built-up area	SOS	y= –0.44x+944.2	0.25	Broadleavf forest	SOS	y= –0.38x+854.2	0.14
	EOS	y= 0.21x-128.2	0.11		EOS	y= –0.3x+896.2	0.10
	LOS	y= 0.65x-1072.5	0.36		LOS	y= 0.08x+61.99	0.01
Arable land	SOS	y= 0.56x-1035.2	0.19	Coniferou forest	SOS	y= –0.65x+1399	0.12
	EOS	y= 0.26x-241.1	0.20		EOS	y= –0.15x+603.9	0.01
	LOS	y= –0.29x+794.1	0.06		LOS	y= 0.51x-795.2	0.04
grassland	SOS	y= –0.18x+449.3	0.05	Mixed forests	SOS	y= –0.51x+1098	0.14
	EOS	y= 0.21x-138.1	0.10		EOS	y= –0.11x+523.3	0.01
	LOS	y= 0.39x-587.4	0.15		LOS	y= 0.40x-575.2	0.05
Sparse Grassland	SOS	y= –0.56x+1207	0.18	Farmland and Natural Vegetation	SOS	y= –0.43x+949.5	0.12
	EOS	y= 0.02x+258.2	0.01		EOS	y= 0.35x-403.8	0.09
	LOS	y= 0.58x-949.1	0.11		LOS	y= 0.78x-1353.3	0.21

Data analysis indicated that the vegetation regrowth period in the middle and lower reaches of the Yangtze River has undergone significant changes over the past two decades. With the exception of cropland ecosystems, which exhibited a delayed trend at a rate of 0.5553 d/a, all other vegetation types in the study area demonstrated varying degrees of advancement in the start of the growing season.

Among the vegetation types, coniferous forest ecosystems exhibited the most significant advancement in the start of the growing season, with an average annual rate of change of –0.6561 d/a and a cumulative advancement of 14.434 days over 22 years. This trend may be attributed to the higher sensitivity of coniferous forests to temperature increases. In contrast, grassland ecosystems displayed only a weak advancement trend, with a rate of change of –0.1826 d/a. The end of the growing season for broadleaf forests, coniferous forests, and mixed coniferous-broadleaf forests showed an advancing trend, with broadleaf forests experiencing the most pronounced advancement at a rate of 0.2927 d/a. Conversely, EOS in urban built-up areas, croplands, grasslands, sparse grasslands, and the mosaic of farmland and natural vegetation exhibited a delaying trend. Among these, the most significant delay was observed in the mosaic of farmland and natural vegetation, with an average annual delay rate of 0.35 d/a, resulting in a cumulative delay of 7.7 days over 22 years.

Observations of the growth cycle across different vegetation types indicate that, except for arable land, all vegetation types exhibit a tendency toward an extended growing period. This lengthening is primarily influenced by two key climatic factors: the advancement of the vegetation greening period and the delay in the end of the growing season.

Arable land exhibited a distinct response pattern, with a slight shortening of the growing season at a rate of 0.2937 d/a. In contrast, vegetation LOS in urban built-up areas, agricultural land, and vegetation mosaics displayed a significant lengthening trend, with rates of 0.6502 d/a and 0.7836 d/a, respectively. Over the past 22 years, this trend resulted in an extension of the growing season by about 14.3044 days in urban built-up areas and 17.2392 days in vegetation mosaics. The observed variability among vegetation types may be attributed to the specific management practices in cropland ecosystems, such as tillage systems, irrigation methods, and crop variety selection. Additionally, these differences highlight the contrast between natural and human-managed ecosystems in their responses to climate change.

### 3.3 Stability analysis

**Figure 6:**
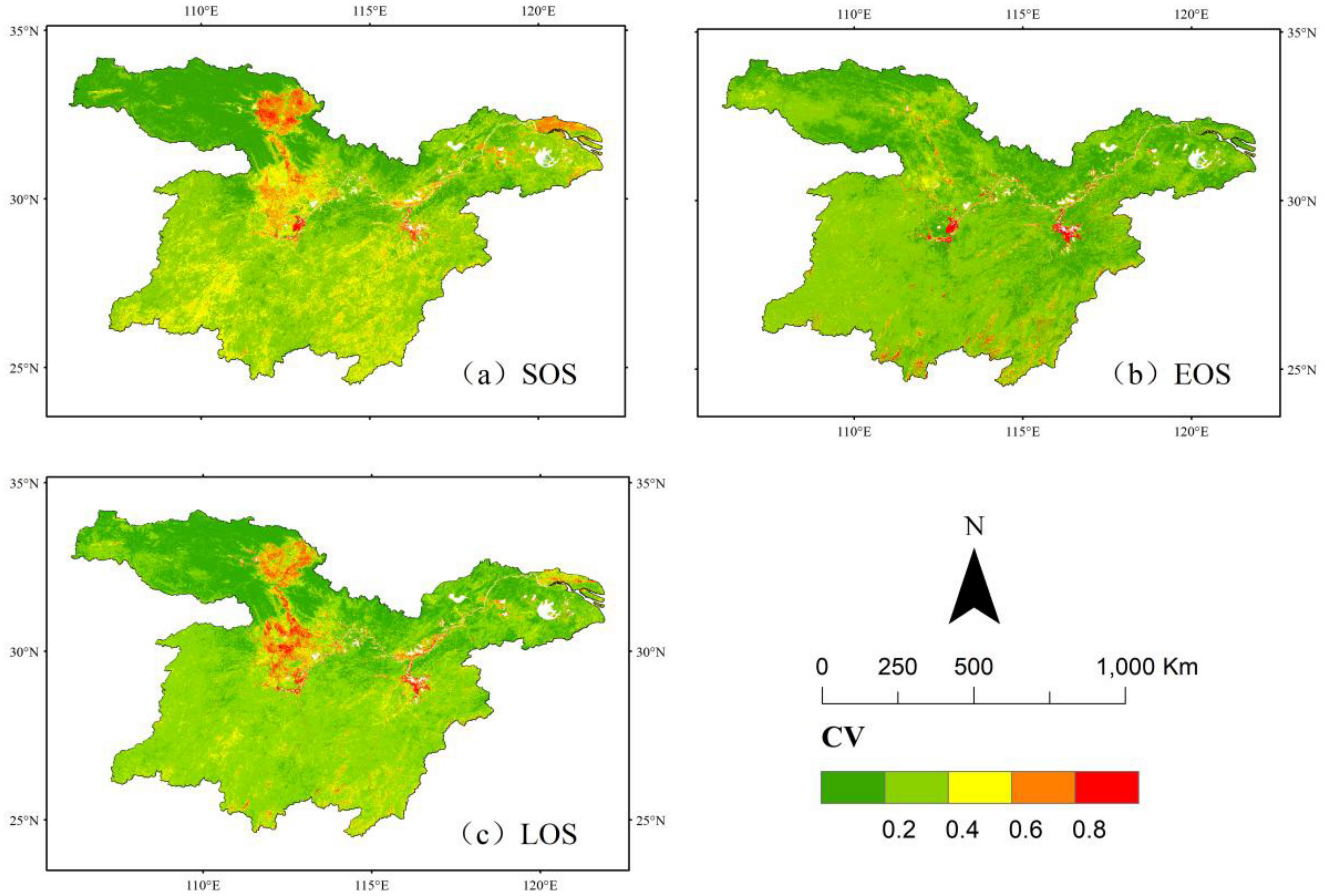
Spatial stability analysis of vegetation phenology in the middle and lower reaches of the Yangtze River. **(a)** Stability analysis of sos. **(b)** Stability analysis of eos. **(c)** Stability analysis of los.

Using the coefficient of variation (CV) method, this study systematically assessed the spatial and temporal fluctuation characteristics of vegetation phenology parameters in the study area from 2001 to 2022. The spatial distribution pattern of these parameters is presented in **Figures 6**.

The average coefficient of variation (CV) for vegetation in the study area was 0.32, exhibiting a trend of gradual stabilization from southeast to northwest. Medium fluctuation accounted for 59.02% of the total area and was predominantly distributed in the southern and eastern regions. Relatively high and high fluctuations were observed mainly in farmland, comprising 4.53% and 0.82% of the area, respectively. The average CV of vegetation EOS was 0.066, indicating primarily low and relatively low fluctuation levels. The proportions of these categories were about 29.85% and 62.33%, respectively, suggesting that vegetation phenology has remained highly stable over the past 22 years.

The characterization of the spatial and temporal variability of the length of the growing season (LOS) in the study area indicated that the average coefficient of variation was 0.15, suggesting a relatively stable overall change. In terms of spatial distribution, low fluctuation areas accounted for 25.44%, while relatively low fluctuation areas comprised 59.02%, collectively covering 84.46% of the study area. This distribution pattern further confirmed the high temporal stability of vegetation growing season length. However, in the central part of the region, vegetation phenology parameters exhibited moderate fluctuation characteristics in 10.49% of the area and relatively high fluctuation in 3.42%. The remaining regions were primarily characterized by low and relatively low fluctuation, indicating a stable vegetation growing season length.

### 3.4 Continuity analysis

**Figure 7:**
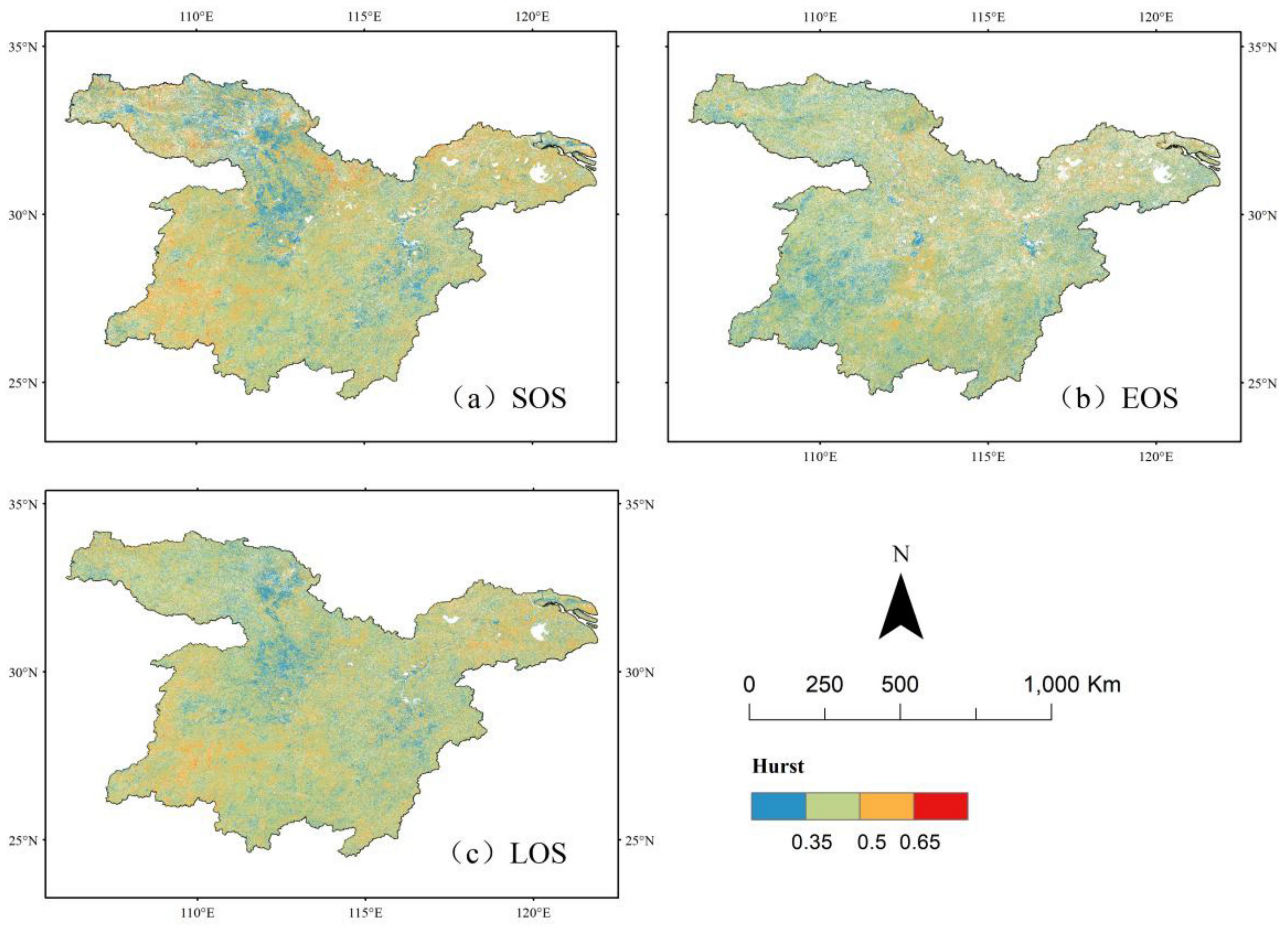
Spatial distribution of vegetation phenology Hurst index in the middle and lower reaches of the Yangtze River. **(a)** Spatial distribution of H values of sos. **(b)** Spatial distribution of H values for eos. **(c)** Spatial distribution of H values of los.

In this study, the Hurst index of vegetation phenology parameters in the middle and lower reaches of the Yangtze River was systematically calculated at an image-by-image metric scale from 2001 to 2022 using R/S analysis to evaluate the persistence characteristics of future vegetation phenology trends. The trend analysis results revealed the spatial and temporal evolution of vegetation phenology in the study area, including its current status, future trends, and historical change processes. The specific spatial distribution pattern is presented in **Figures 7**. The analysis indicated that the Hurst index values for the start of the growing season (SOS), the end of the growing season (EOS), and the length of the growing season (LOS) ranged from 0.35 to 0.5. Their respective spatial distribution ratios were 58.06%, 63.15%, and 63.82%, covering the entire study area. These quantitative results suggest that the changes in vegetation climatic parameters exhibit significant anti-continuity, meaning that future trends are likely to develop in the opposite direction of historical changes. This implies a potential delay in the start of the growing season and an advancement in its end, ultimately leading to a shortened growing season. Such synergistic variations in climatic parameters may exert a significant influence on the regional ecosystem’s carbon cycle.

**Figure 8:**
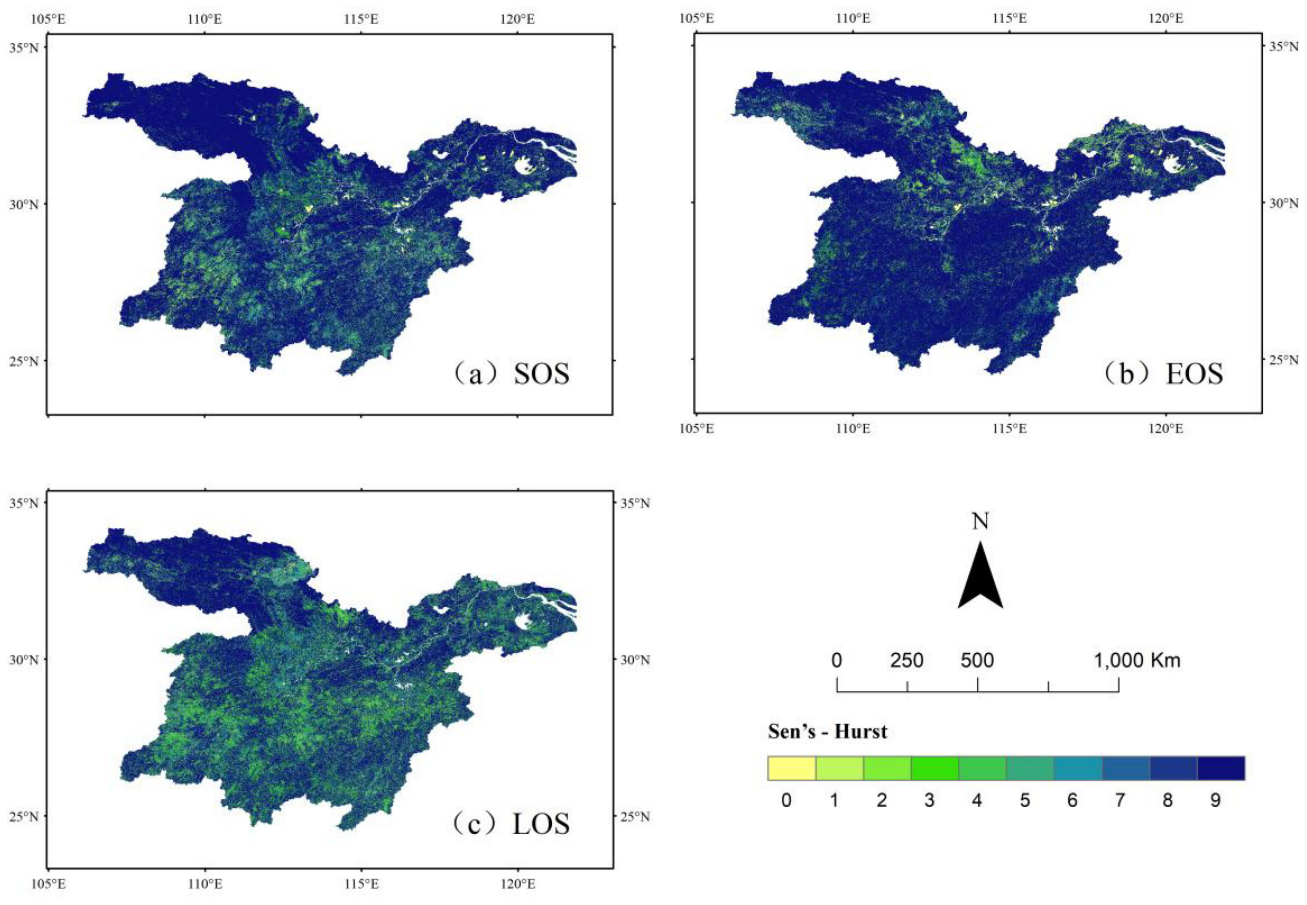
Spatial distribution of sustainability of vegetation phenology changes in the middle and lower reaches of the Yangtze River from 2001 to 2020. **(a)** Future trend analysis of sos. **(b)** Future trend analysis of eos. **(c)** Future trend analysis of los.

The proportion of image elements with Hurst index values between 0.5 and 0.65 was 24.56% for SOS, 16.48% for EOS, and 20.25% for LOS. The spatial distribution of vegetation SOS and LOS was primarily concentrated in the southeastern part of the study area, while EOS was mainly distributed in the southern region, with all exhibiting a more dispersed pattern. This suggests that these areas are likely to maintain the historical trends of vegetation phenology parameters, with SOS advancing, EOS delaying, and LOS prolonging, as indicated by the overlay analysis of Sen’s Slope and the Hurst index. As shown in **Figures 8**, the future evolution trends of vegetation phenology remain consistent with past patterns, with 75.28% of the study area expected to experience continued advancement of the greening period (SOS), 82.15% expected to show further delays in the yellowing period (EOS), and 59.16% expected to exhibit an extended growing season length (LOS).

**Table 3** indicates that the future vegetation rejuvenation period (SOS) in the middle and lower reaches of the Yangtze River is expected to continue advancing, with 19.53% of the region exhibiting this trend. In terms of spatial distribution, the west-central and southern parts of the Yangtze River, predominantly covered by grasslands, demonstrate a consistent advancement at the start of the growing season. This pattern may be attributed to the higher sensitivity of grassland ecosystems to climate change and highlights the substantial differences in the response of various vegetation types to climate warming. Areas exhibiting a continuous delay in SOS are primarily cultivated land. Additionally, regions that previously experienced a delayed SOS but are projected to advance in the future are also concentrated in the south-central part of the study area, predominantly within grassland ecosystems.

**Table 3:** Future trends of vegetation phenological parameters in the middle and lower reaches of the Yangtze River.

Pixel	Percentage(%)			Growing trend	Future trends		
	Sos	Eos	Los		Sos	Eos	Los
1	3.20	0.03	0.04	Strong persistent degradation	advance	defer	lengthen
2	3.39	0.95	1.66	Weak persistent degradation			
3	0.97	1.45	3.87	Anti-Strength Sustainability Improvement	Past ahead but future delayed	The past is delayed but the future is ahead	The past is lengthening but the future is shortening
4	3.08	5.27	17.73	Anti-Weakness Sustainability Improvement			
5	10.37	3.69	5.96	Antiweak persistent degradation	The past is delayed but the future is ahead	Past ahead but future delayed	The past is shorter but the future is longer
6	2.37	1.06	1.91	Anti-strong persistent degradation			
7	1.20	1.59	6.11	Weak persistent improvement	defer	advance	curtail
8	0.06	0.06	0.14	Strong Continuous Improvement			
9	75.28	82.15	59.16	unchanged	unchanged	unchanged	unchanged

The region exhibiting a continuous delay in vegetation EOS accounted for 5.73% of the study area and was primarily distributed in the southeastern and northwestern regions, which were predominantly woodlands. In contrast, areas showing a continuous advancement of EOS covered 8.37% of the region, mainly concentrated in the north-central and southwestern areas, where cropland was the dominant vegetation type. Vegetation LOS was primarily characterized by a persistent shortening trend, affecting 27.85% of the study area. Among these, reverse persistence was the most prevalent, comprising 21.60%, with 17.73% classified as reverse weak persistence and 3.87% as reverse strong persistence. However, cultivated land exhibited a trend of persistent LOS extension (**Figure 8**).

The primary trends of future vegetation phenology evolution in the middle and lower reaches of the Yangtze River include the persistent advancement of SOS and EOS, with the most pronounced changes observed in SOS. Additionally, the trend of EOS advancing persistently, along with a shift from previous advancements to future delays, is becoming increasingly evident.

### 3.5 Response of vegetation phenology to climate change

#### 3.5.1 Spatial distribution of climate change

**Figure 9:**
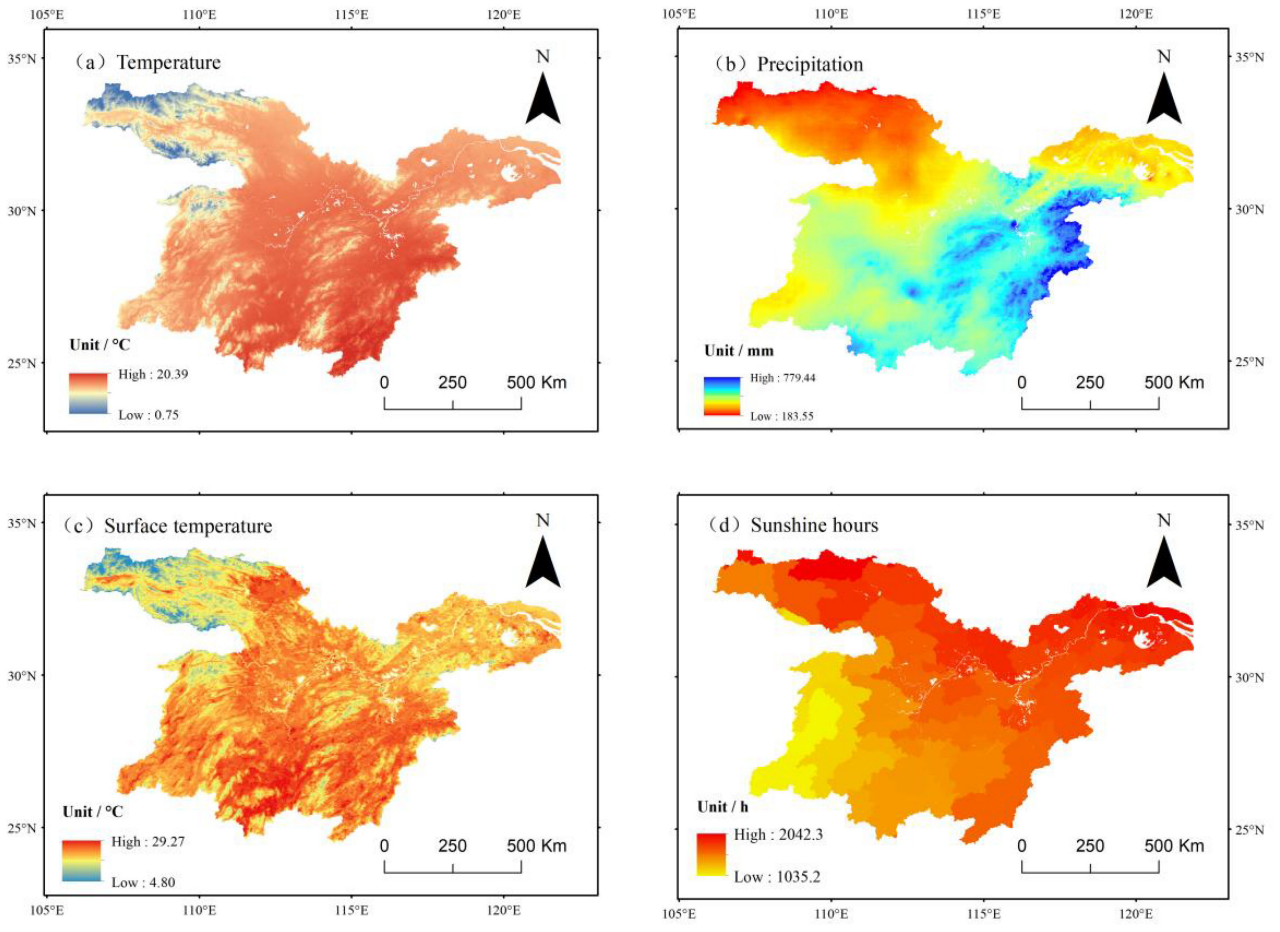
Spatial distribution of climate factors in the middle and lower reaches of the Yangtze River from 2001 to 2020. **(a)** Annual average temperature. **(b)** Average annual precipitation. **(c)** Annual average surface temperature. **(d)** Average annual cumulative sunshine hours.

The study examines the multi-year spatial variations of key climate factors, including temperature, precipitation, surface temperature, and average annual cumulative sunshine hours, in the middle and lower reaches of the Yangtze River. As shown in **Figures 9**, an analysis based on meteorological data from 2001 to 2020 reveals distinct climate gradient characteristics in the study area. The annual mean temperature varies between 0.75°C and 20.39°C, following a clear latitudinal zonation pattern, where temperature increases as latitude decreases. Precipitation exhibits significant spatial variability, ranging from 185.33 mm to 779.44 mm, with a distribution pattern characterized by a decline from southeast to northwest. The annual average surface temperature is slightly higher than the annual average air temperature, ranging from 4.8°C to 29.27°C, and shows a decreasing trend with increasing latitude. The annual average cumulative sunshine hours range from 1035.2 to 2042.3 h, with a general tendency for sunshine duration to increase as latitude increases.

#### 3.5.2 Response of vegetation return period (SOS) to climatic factors

**Figure 10:**
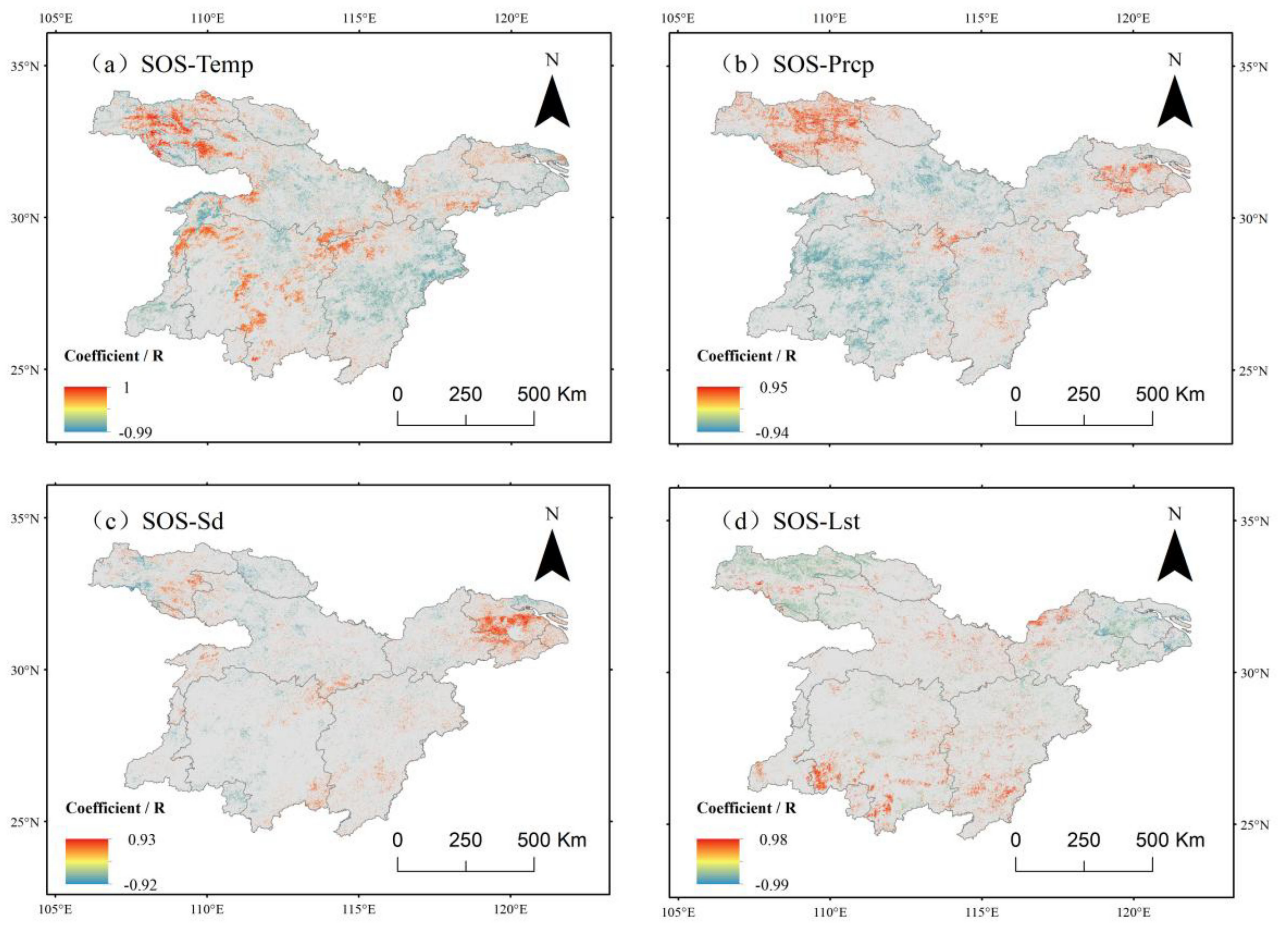
Correlation between sos and climate factors in the middle and lower reaches of the Yangtze River. **(a)** Correlation analysis between sos and mean air temperature. **(b)** Correlation analysis between sos and average precipitation. **(c)** Correlation analysis between sos and mean accumulated solar insolation hours. **(d)** Correlation analysis between sos and average surface temperature.

The image-by-image meta-analysis of SOS during vegetation regrowth in the middle and lower reaches of the Yangtze River was conducted using Pearson correlation analysis. The spatial distribution of correlation coefficients that passed the significance test (P<0.05) is presented in **Figure 10**.

Correlation analysis with air temperature (**Figure 10a**) revealed that in the northwestern region, a significant delay effect was observed between temperature increases and the vegetation rejuvenation period, which may be attributed to the unique temperature threshold effect in high-altitude areas. In terms of quantitative characteristics, spatial units exhibiting a negative correlation accounted for 55.16% of the study area, with the eastern region displaying a particularly significant negative correlation at a proportion of 10.21%. This spatial distribution pattern suggests that in the eastern region, the vegetation rejuvenation period tends to advance as temperature increases. This response pattern is likely associated with the lower altitude and higher cumulative temperature conditions in the eastern part of the study area.

In terms of spatial distribution, the correlation between the start of the growing season (SOS) and precipitation (**Figure 10b**) exhibited a significant negative correlation in 58.45% of the study area. Positive correlation areas, accounting for 41.55%, were primarily concentrated in the northwestern, northeastern, and southeastern regions, where increased precipitation was associated with a delayed vegetation rejuvenation period. Notably, in the northwestern region, the vegetation rejuvenation period showed a significant positive correlation (5.66%) with temperature. In contrast, the southwestern and north-central parts of the study area displayed a strong negative correlation with precipitation, with about 10% of the pixels exhibiting a significant negative correlation. These variations suggest that changes in climatic factors in these regions follow an opposite trend to the vegetation greening period, indicating the presence of distinct phenological response mechanisms.

Correlation analysis with sunshine hours (**Figure 10c**) indicated a weak negative correlation between vegetation SOS and sunshine duration, with a relatively uniform spatial distribution. Positive correlation areas accounted for 47.47% of the study region and were scattered, with a distinct concentration in the northeastern part, covering 4.23% of the area. In contrast, negative correlation areas comprised 51.53% and were primarily distributed in the western region of the central part of the study area.

The correlation analysis with surface temperature (**Figure 10d**) indicated that vegetation SOS exhibited a predominantly positive correlation in the southern region, accounting for 64.44% of the study area. This suggests that in this region, the start of the growing season tended to be delayed with increasing surface temperature. In contrast, a negative correlation was observed in the northern region, covering 35.56% of the area, indicating that SOS gradually advanced with rising surface temperature. Both correlations passed the significance test, with 4.11% of the positive correlation and 6.88% of the negative correlation being statistically significant.

It can be concluded that vegetation SOS generally exhibited a negative correlation with air temperature, precipitation, sunshine hours, and surface temperature.

#### 3.5.3 Response of vegetation obsolescence (EOS) to climatic factors

**Figure 11:**
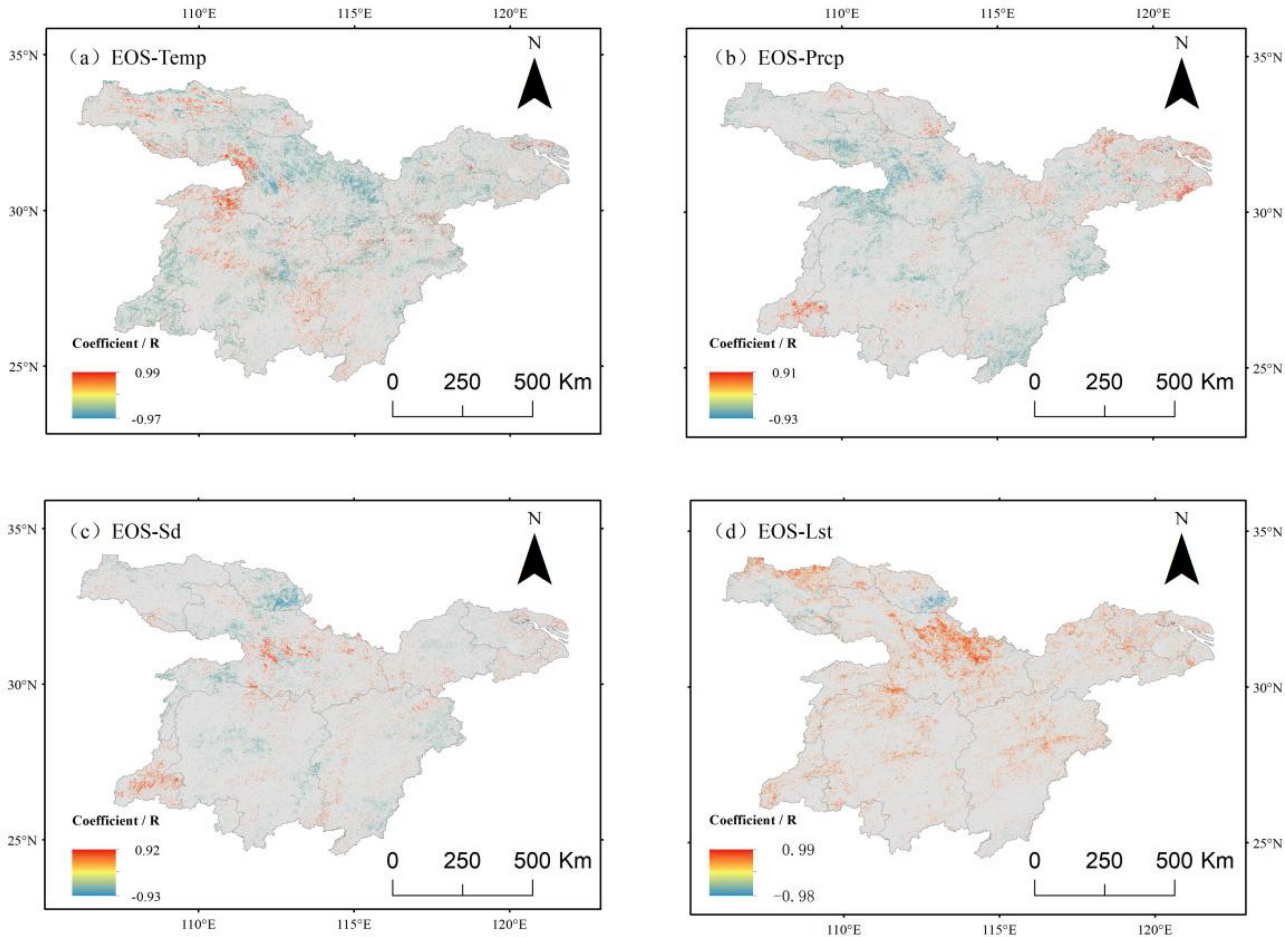
Correlation between eos and climate factors in the middle and lower reaches of the Yangtze River. **(a)** Correlation analysis between eos and mean air temperature. **(b)** Correlation analysis between eos and mean precipitation. **(c)** Correlation analysis between eos and mean cumulative sunshine hours. **(d)** Correlation analysis between eos and mean surface temperature.

Regarding temperature, the analysis results presented in **Figure 11a** indicate that vegetation EOS and air temperature in the study area predominantly displayed a negative correlation, with 58.61% of the area showing this trend. Among these, 11.79% of the image elements passed the significance test, primarily concentrated in the northwestern and southern regions, where vegetation EOS tended to advance with increasing air temperature.

Correlation analysis with precipitation (**Figure 11b**) revealed a significant negative correlation between vegetation EOS and precipitation in the northwest and southeast regions, with negatively correlated areas accounting for 61.13% of the total study area. Among these, 9.48% of the regions exhibited a statistically significant negative correlation. In contrast, positively correlated regions comprised 38.87% of the area. The spatial distribution pattern showed that positive correlation areas were mainly located in the northeast and southwest regions, suggesting that increased precipitation may contribute to a delayed end of vegetation growth.

The correlation between vegetation EOS and sunshine hours (**Figure 11c**) was primarily observed in the southern, northwestern, and northeastern regions, exhibiting a weak negative correlation with a likelihood ratio of 55.30%. In the northwestern region, the negative correlation coefficient was more pronounced, with a likelihood ratio of 5.5%. Conversely, the positive correlation areas were mainly distributed in the north-central and southwestern regions, accounting for 44.70% of the study area. In the southwestern region, a stronger positive correlation was detected, indicating that an increase in sunshine hours contributed to the delayed trend of vegetation EOS in this region.

Correlation analysis with surface temperature (**Figure 11d**) revealed that vegetation EOS exhibited a significant positive correlation with surface temperature. The proportion of positively correlated areas was 73.36%, with 8.05% of these regions passing the significance test. This suggests that rising surface temperature was associated with a notable delay in vegetation EOS across the study area.

These findings indicate that vegetation EOS demonstrated a strong positive correlation with surface temperature. The influence of surface temperature on vegetation EOS contrasts with the effects of air temperature, precipitation, and sunshine hours. Specifically, an increase in surface temperature leads to a delayed EOS, whereas increases in air temperature, precipitation, and sunshine hours tend to advance EOS.

#### 3.5.4 Vegetation growing season length (LOS) response to climate factors

**Figure 12:**
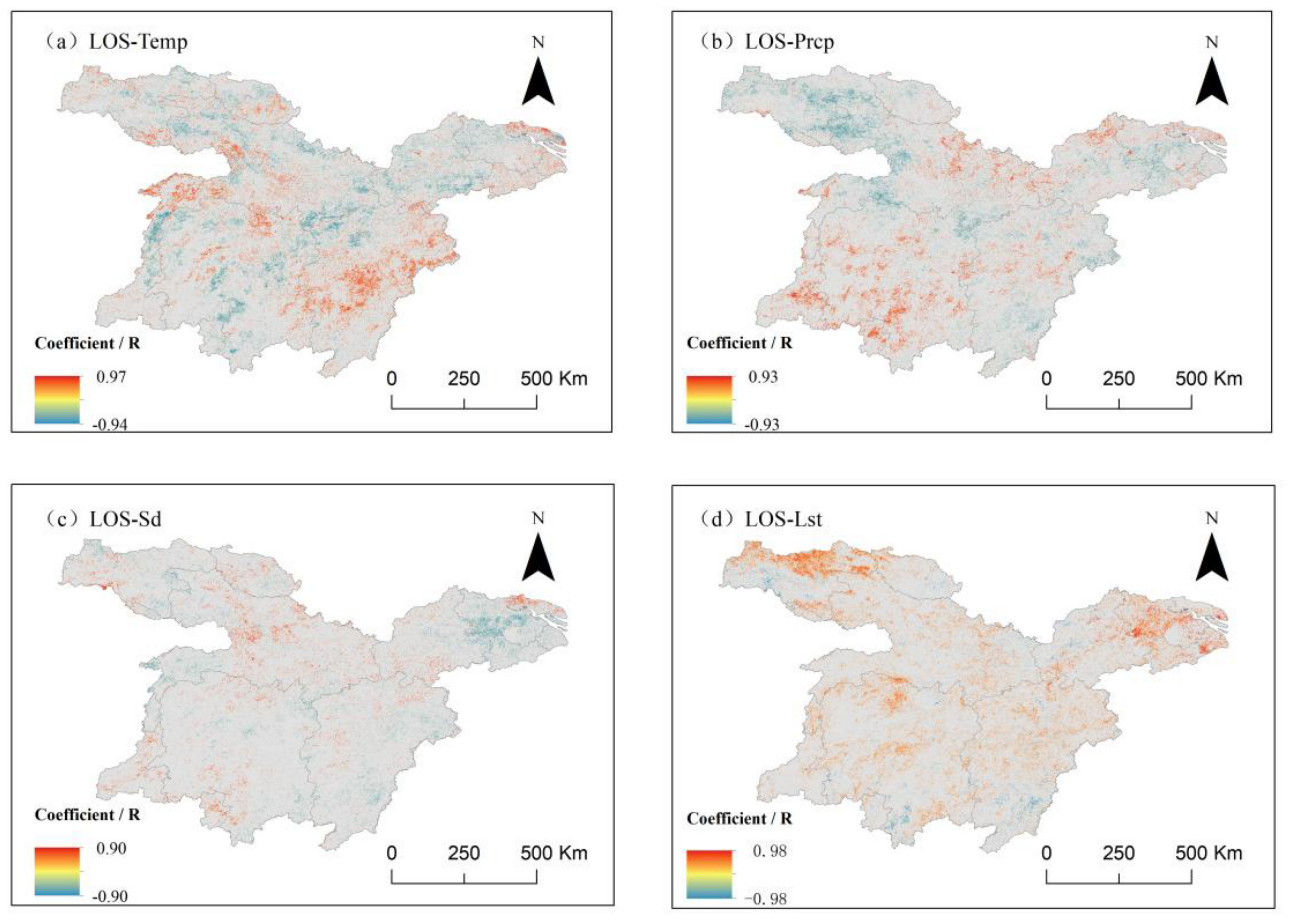
Correlation between los and climate factors in the middle and lower reaches of the Yangtze River. **(a)** Correlation analysis between los and average air temperature. **(b)** Correlation analysis between los and average precipitation. **(c)** Correlation analysis between los and mean accumulated sunshine hours. **(d)** Correlation analysis between los and average surface temperature.

The correlation coefficients between vegetation growing season length (LOS) and climate factors (**Figure 12**) indicate that the correlation between LOS and temperature exhibited a weak negative trend, with negatively correlated pixels accounting for 51.58% of the study area. The proportion of positively correlated pixels was 48.42%, with both distributions being spatially dispersed. The positive correlation between LOS and temperature was more pronounced in the southeast and northwest regions, covering 8.73% of the pixels. Conversely, a significant negative correlation was observed in the central region, also covering 8.73% of the pixels, suggesting that increased temperature contributed to a shortened LOS in these areas.

Correlation analysis with precipitation (**Figure 12b**) revealed an overall weak positive correlation between LOS and precipitation, with positively correlated pixels comprising 50.15% and negatively correlated pixels 49.85%. The positive correlation was primarily concentrated in the southwestern region, where LOS lengthened with increased precipitation, with the most significant positive correlation observed in 5.76% of the pixels. The negative correlation area was mainly located in the northwestern region, where 7.31% of the pixels exhibited a significant negative correlation, indicating that increased precipitation contributed to a reduction in LOS.

The results of correlation analysis with sunshine hours (**Figure 12c**) showed a slight difference between the positive and negative correlations, accounting for 48.43% and 51.57% of the total image elements, respectively, indicating an overall weak negative correlation between LOS and sunshine hours.

The correlation with surface temperature (**Figure 12d**) demonstrated that LOS was predominantly positively correlated with surface temperature, meaning that LOS tended to increase as surface temperature rose. The proportion of positively correlated pixels was 72.16%, with an even spatial distribution across the study area. A significant positive correlation was detected in the northeastern and northwestern regions, where 10.14% of the pixels showed a strong relationship between increasing surface temperature and prolonged LOS.

The correlations between vegetation LOS (the length of the growing season) and temperature, precipitation, and sunshine hours were relatively balanced between positive and negative values. In the central region, a significant negative correlation was observed between LOS and air temperature, whereas in the southwestern region, LOS exhibited a strong positive correlation with precipitation. In the northeastern region, LOS showed a significant negative correlation with sunshine hours. Across most of the study area, LOS displayed a positive correlation with surface temperature, indicating that an increase in surface temperature contributed to the lengthening of the vegetation growing season.

## 4 Discussion

### 4.1 Characteristics of changes in vegetation phenology

On the temporal scale, vegetation phenology in the middle and lower reaches of the Yangtze River exhibited a clear trend of advancement in the start of the growing season (SOS) and a delay in the end of the growing season (EOS), aligning with findings from previous studies (Cui Hongjing, 2024; Han et al., 2024; Yang et al., 2022, Yang et al., 2024; Zhu et al., 2024). The results consistently indicated an advancing trend for SOS and a delaying trend for EOS. Since 2019, SOS has shown a delayed pattern, peaking in 2021, which resulted in the shortest growing season length that year. The temperature increase in 2019 may have contributed to the advancement of vegetation rejuvenation, while the subsequent rise in precipitation from 2019 onward was also significantly correlated with these changes.

On the spatial scale, vegetation phenology was found to be highly sensitive to environmental changes, particularly near urban fringes, where intensive human activities led to environmental modifications that significantly impacted vegetation phenology (Yuan et al., 2023). The SOS of farmland and natural vegetation mosaic areas differed from that of urban built-up areas by 2.64 d/a, with the most pronounced difference observed in EOS, reaching 7.7 d/a, which was consistent with the findings of Haiyong Ding (Ding et al., 2020). Within urban built-up areas, surface temperature exhibited a significant increasing trend, leading to notable changes in the phenology of farmland, while other vegetation types experienced relatively minor phenological shifts.

Observations indicate that the primary driver of SOS advancement and EOS delay over the past 22 years has been the increase in surface temperature, which has emerged as a key factor contributing to phenological variations. The response of vegetation phenology to climate change exhibits significant spatial heterogeneity, influenced by interactions among multiple environmental factors.

Although research has revealed the roles of air temperature, precipitation, surface temperature, and hours of sunshine in vegetation phenology, how these factors work together remains understudied. Environmental factors, such as photoperiod, albedo, evapotranspiration, and water availability, influence vegetation phenology. On the Tibetan Plateau, vegetation greening attenuates surface warming by increasing evapotranspiration (ET). In the tropics and subtropics, the evaporative cooling effect of increased vegetation activity on surface temperature is usually more significant than the evaporative warming effect. Enhanced vegetation activity (e.g., earlier or longer growing seasons) may reduce surface albedo (e.g., reduced snow cover or increased vegetation cover), resulting in more solar radiation being absorbed and higher surface temperatures, which can further promote vegetation growth. Studies have shown that enhanced vegetation activity in temperate grasslands significantly increases spring and fall temperatures through albedo reduction (Shen et al., 2022).

### 4.2 Influence of vegetation cover type on phenology

From 2001 to 2022, vegetation SOS, except for cultivated land, exhibited an advancing trend, with varying magnitudes of change. Qiu Tong stated that different land cover types exhibit distinct phenological characteristics (Qiu et al., 2017), which aligns with the findings of this study. Variations in vegetation cover types influence the temporal changes in phenology, as they determine the magnitude of SOS and EOS values at different time points. Vegetation cover in urban built-up areas is heavily affected by human activities, with LOS extending at a rate of 0.65 d/a. Some studies have indicated that land cover changes have a more substantial impact on the physical climate than climatic factors alone. Urban expansion, land conversion, and ecological policies such as farmland reforestation have significantly altered the vegetation growth environment, indirectly contributing to interannual variations in the physical climate. However, since these land cover changes represent a relatively small portion of the overall study area, the results remain representative of regional climatic variations. It is important to acknowledge that a broader range of vegetation cover types exists beyond the eight categories examined in this study. For instance, shrubland, which occupies a relatively small area, was not considered in the analysis.

Zhu et al. elucidated the dynamic reflection of urbanization in phenology in their experiments, revealing the complex impacts of urbanization on vegetation phenology. Yang analyzed the characteristics of phenology changes in the Yangtze River Delta from three latitudes: time, planar space and elevation. Therefore, most of the previous studies chose urban agglomerations to explore the effects of urbanization on the spatial and temporal changes of vegetation phenology. In this paper, we focus on the middle and lower reaches of the Yangtze River, as a key ecological transition zone in China, to explore the response of its vegetation phenology to climate change, which makes up for the lack of regional specificity in previous Northern Hemisphere-scale studies. Richardson et al. (Richardson et al., 2013) proposed that fall phenology is mainly regulated by air temperature and photoperiod, but they did not incorporate the influence of land surface temperature (LST). Their modeling framework emphasized the driving role of atmospheric temperature on vegetation senescence but did not consider the independent effect of surface energy exchange. The study (Yuan et al., 2024) found that nighttime temperature had a more substantial impact on the advance of the greening stage than daytime temperature and that regulating the senescence stage by precipitation and solar radiation was time-scale dependent. Piao pointed out that vegetation growth in arid zones was subject to the antagonistic effects of air temperature and soil moisture but did not explore the contrast between LST and air temperature. Therefore, the study in this paper found the opposite effects of LST and air temperature on EOS, which may extend the scope of antagonistic mechanisms.

### 4.3 Uncertainty analysis

This study examines the response of vegetation phenology to air temperature, precipitation, surface temperature, and insolation. The findings indicate that increasing surface temperature results in the advancement of SOS, the postponement of EOS, and the lengthening of LOS, which differs from the effects of air temperature and other climatic factors. Climatic change is a prolonged and complex process influenced by multiple environmental conditions. In addition to the climatic factors selected for the experiment, light radiation, cumulative temperature, and carbon dioxide also significantly affect phenology (Xiang et al., 2024). During urbanization, alterations in vegetation cover types may influence vegetation phenology (Zhang et al., 2020), while various pollution factors, including light and air pollution, can impact vegetation phenology by affecting photosynthesis (Xia et al., 2017).

Additionally, research has indicated that the influence of human activities on vegetation and climate has become increasingly evident. In agricultural areas, drought- and flood-tolerant seeds or crops are selectively bred to enhance yields. In urban environments, resilient trees or shrubs are chosen for street planting, with maintenance practices such as pruning and watering conducted periodically. To extend the growth cycle of plants, specific artificial methods are employed to modify environmental conditions, thereby affecting the vegetation climate (Hu et al., 2024).

The data and methods used in this study introduce certain uncertainties. Due to the lack of direct observational data, the extracted phenology results were validated using indirect verification methods. As shown in **Table 4**, the results for the middle and lower reaches of the Yangtze River align with findings from previous studies. However, variations in values may arise due to differences in study areas and methodological approaches. The Enhanced Vegetation Index (EVI) dataset was utilized to analyze vegetation phenology, but its temporal resolution is limited. While the MOD13Q1 dataset used in this study offers a spatial resolution of 250 m, its 16-day temporal resolution may present challenges in accurately capturing vegetation phenology dynamics over shorter periods.

**Table 4:** Comparison of the optimal phenological dataset in this study with previous findings.

Study area	Study time	Extraction method	SOS	EOS	Data sources
Yangtze River	2001–2019	curvature change extremum method	-0.17d/a	0.48d/a	MCD12Q2
Delta [Yang et al.]	2001–2020	Maximum rate of change method	-0.41d/a	0.16d/a	MOD13Q1
Yangtze River	1990–2020	Dynamic thresholding	-0.30d/a	0.80d/a	MOD13Q1
Delta [Zhu et al.]	2002–2020	dynamic thresholding	-0.58d/a	0.08d/a	MOD13Q1
Three gorges reservoir area [Cui et al.]	2001–2019	dynamic thresholding	-0.12d/a	0.16d/a	MOD13A2

Future research will incorporate high-resolution temporal and spatial imagery to enhance the analysis. Given the significant impact of human activities on vegetation phenology, subsequent studies could focus on urban areas to explore their role in modifying phenological patterns. Additionally, this study did not account for annual changes in vegetation cover types, nor did it consider other influential factors such as light radiation, cumulative temperature, and carbon dioxide. Future research will address these gaps to further refine and improve the methodology.

## 5 Conclusions

To advance phenology and enhance the understanding of global ecological responses, we extracted vegetation phenology, including the start of the growing season, the end of the growing season, and the length of the growing season, based on the Enhanced Vegetation Index evi, and explored its spatial and temporal variability in the middle and lower reaches of the Yangtze River and its relationship with climate. The findings indicate that:

1. From 2001 to 2022, the study areas, the start of the growing season (SOS) advanced at a rate of 0.29 days per year, primarily occurring between the 62nd and 97th days. The end of the growing season (EOS) was delayed by 0.26 days per year, mainly concentrated between the 281st and 315th days. The length of the growing season (LOS) increased by 0.56 days per year, with values generally ranging between the 206th and 243rd days, exhibiting noticeable interannual fluctuations.
2. Among different vegetation types, cultivated land showed the most significant SOS delay and LOS shortening, with rates of 0.5553 d/a and 0.2937 d/a, respectively. The LOS in urban built-up areas, farmland, and vegetation mosaics showed a pronounced lengthening trend, extending by 14.3044 days and 17.2392 days over 22 years. Broadleaf forests exhibited the most notable EOS advancement, with a rate of 0.2927 d/a.
3. The average coefficient of variation (CV) for vegetation SOS in the middle and lower reaches of the Yangtze River was 0.32, indicating an overall stable trend. The average CV for EOS was 0.066, reflecting high stability over the past 22 years. The average CV for LOS was 0.15, showing relative stability except for higher fluctuations observed in some arable land and farmland areas.
4. The Hurst index analysis of SOS, EOS, and LOS suggested that the overall trend of vegetation change in the middle and lower reaches of the Yangtze River is expected to be opposite to the patterns observed over the past 22 years, indicating a weak tendency toward delayed SOS, advanced EOS, and shortened LOS. In contrast, in the southern part of the study area, SOS, EOS, and LOS are projected to show trends of advancement, delay, and lengthening, respectively.
5. Air temperature, precipitation, surface temperature, and sunshine hours in the middle and lower reaches of the Yangtze River were all correlated with vegetation SOS. A negative correlation was observed between SOS and air temperature, precipitation, sunshine hours, and surface temperature. EOS exhibited a significant negative correlation with air temperature, precipitation, and sunshine hours, while it showed an opposite trend with surface temperature. Additionally, LOS demonstrated weak negative correlations with air temperature, precipitation, and sunshine hours, whereas it was positively correlated with surface temperature in most areas, suggesting that an increase in surface temperature contributes to the prolongation of LOS.

## Data Availability

The datasets presented in this article are not readily available because the data that support the findings of this study are not publicly available due to reason, e.g., confidentiality agreements, privacy restrictions, or proprietary concerns. However, summary statistics and processed data are available upon reasonable request. Requests to access the datasets should be directed to ZS,13085505060@163.com.
